# Nutritional Approaches in Autism Spectrum Disorder: A Scoping Review

**DOI:** 10.1007/s13668-025-00655-y

**Published:** 2025-04-22

**Authors:** Ebru Ozler, Nevin Sanlier

**Affiliations:** Department of Nutrition and Dietetics, School of Health Sciences, Ankara Medipol University, 06050 Altındağ, Ankara, Turkey

**Keywords:** Autism spectrum disorder, Gluten, Free, Casein, Free diet, Ketogenic diet, Probiotics, Gut, Brain axis

## Abstract

**Purpose of Review:**

This review was conducted to discuss the etiology of autism in the light of current information, to draw attention to the fact that defects in different biological mechanisms cause autism, and to examine the effectiveness of dietary interventions and supplements in relieving ASD symptoms.

Recent Findings.

Autism spectrum disorder (ASD) is an extremely heterogeneous condition characterized by delays in reciprocal social interaction and communication skills, stereotyped behaviors, and a narrowed range of interests and limited activities. Comorbid conditions such as cognitive impairment, epilepsy, psychiatric diseases, and behavioral symptoms such as impaired social communication, repetitive behaviors, lack of interest in the environment, nutritional disorders, gastrointestinal diseases and abnormal (dysbiotic) states, sleep disorders, and dysmorphism are frequently encountered in individuals with ASD. Although nutrition is one of the environmental factors affecting ASD, it can also be effective in alleviating the behavioral and gastrointestinal symptoms of ASD. Various dietary models (GFCF diet, low glycemic index diet, ketogenic diet, specific carbohydrate diet, Mediterranean diet, GAPS, Feingold, Candida body ecology, allergy elimination diets, etc.) and supplements (vitamin D, polyunsaturated fatty acids, probiotics and prebiotics, phytochemicals) can be used to alleviate symptoms in individuals with ASD.

**Summary:**

The effectiveness and reliability of dietary interventions in individuals with ASD are a matter of significant debate, and the evidence for these practices is limited. Furthermore, there is no consensus on establishing an ideal nutritional model for individuals with ASD.

**Supplementary Information:**

The online version contains supplementary material available at 10.1007/s13668-025-00655-y.

## Introduction

Autism spectrum disorder (ASD) is characterized by repetitive and restricted behaviors and deficits in social communication [1]. DSM- 5 criteria are used to easily diagnose ASD. DSM- 5 defines ASD according to degrees of severity based on deficits in social communication and restricted and repetitive behaviors [2–4]. According to data from the National Center for Health Statistics, it has been suggested that 1 in 36 children may have autism spectrum disorder [5]. WHO reports that 1 in every 100 children is diagnosed with ASD [6]. According to estimates from the Centers for Disease Control and Prevention’s Autism and Developmental Disability Monitoring Network, an average of 1 in 44 children is diagnosed with ASD [4]. This rate is thought to be the same across all racial, ethnic, or socioeconomic backgrounds; however, gender differences exist. ASD is four times more common in boys than girls [7] and it incurs significant costs for managing or supporting individuals with ASD, whether children or adults [8].

**Fig. 1:**
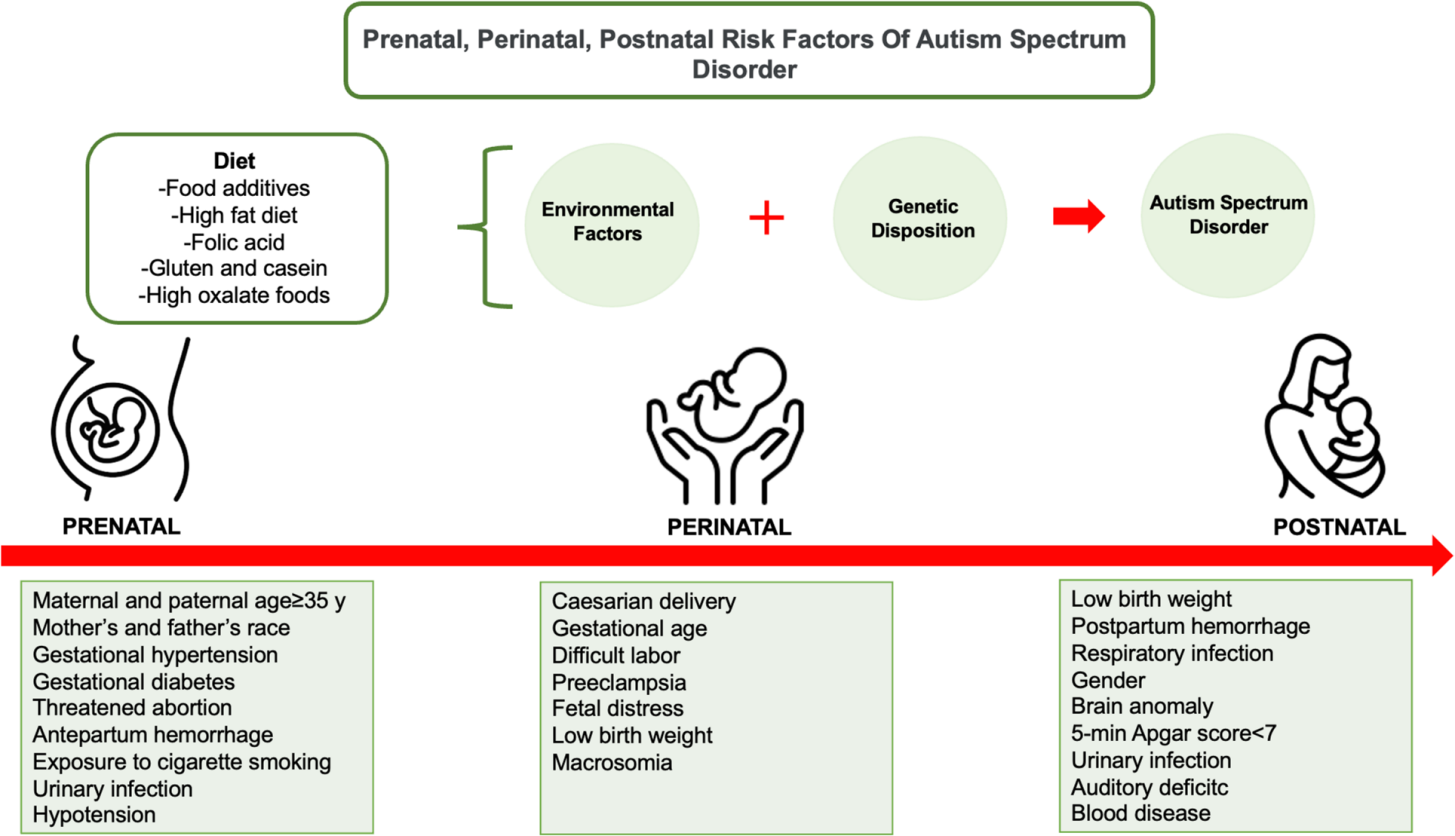
Prenatal, perinatal, postnatal risk factors of autism spectrum disorder [11, 13–15]

Although the exact cause of ASD is not fully known, the most widely accepted causal hypothesis is that it arises from the interaction of multiple factors. The underlying causes of autism include genetic predisposition, epigenetics, neurological factors, metabolic factors, psychosocial factors, prenatal/postnatal factors, mitochondrial dysfunction, cellular stress, environmental and immunological-inflammatory factors, and the interactions of these factors with each other [9, 10]. These are thought to lead to dysfunctional neuronal pathways, abnormal synaptogenesis, neurotransmitter imbalance and neuronal connectivity [11]. The etiology of ASD is complex and still not fully understood [12]. Prenatal, perinatal, and postnatal risk factors in the etiology of ASD are shown in Fig. 1 [12, 14–16]. It is highlighted that generally some factors related to the mother’s nutrition during pregnancy (such as folic acid and fatty acids) may be effective in the development of ASD [12]. In autism spectrum disorder (ASD), the gut-brain axis theory suggests that the nutritional program extending from early period of life to pregnancy may affect cognitive function and cause ASD in genetically susceptible individuals. Even though the impact of the microbiota-gut-brain axis in ASD has been frequently discussed, people have paid a great interest in nutritional interventions [13].

This review was conducted to discuss the etiology of autism in the light of current information, to draw attention to the fact that defects in different biological mechanisms cause autism, and to examine the effectiveness and side effects of dietary interventions applied in alleviating ASD symptoms, their applicability and dietary supplements.

## Nutritional Approaches in Autism Spectrum Disorder

Many children with ASD may prefer to consume only certain foods and may have food selectivity [17]. A study reported that 30–84% of children with ASD had difficulties in food intake [18]. Zinc, calcium, iron, selenium, iodine, methyl-cobalamin, vitamin A, thiamine, pantothenic acid and niacin are the most common nutrient deficiencies in individuals with ASD [19]. Individuals with ASD suffer from many gastrointestinal system complaints such as bloating, abdominal pain, constipation, chronic diarrhea, leaky gut syndrome and gastroesophageal reflux [17]. Gastrointestinal complaints are observed in 9% to 91% of individuals with ASD [20].

**Fig. 2:**
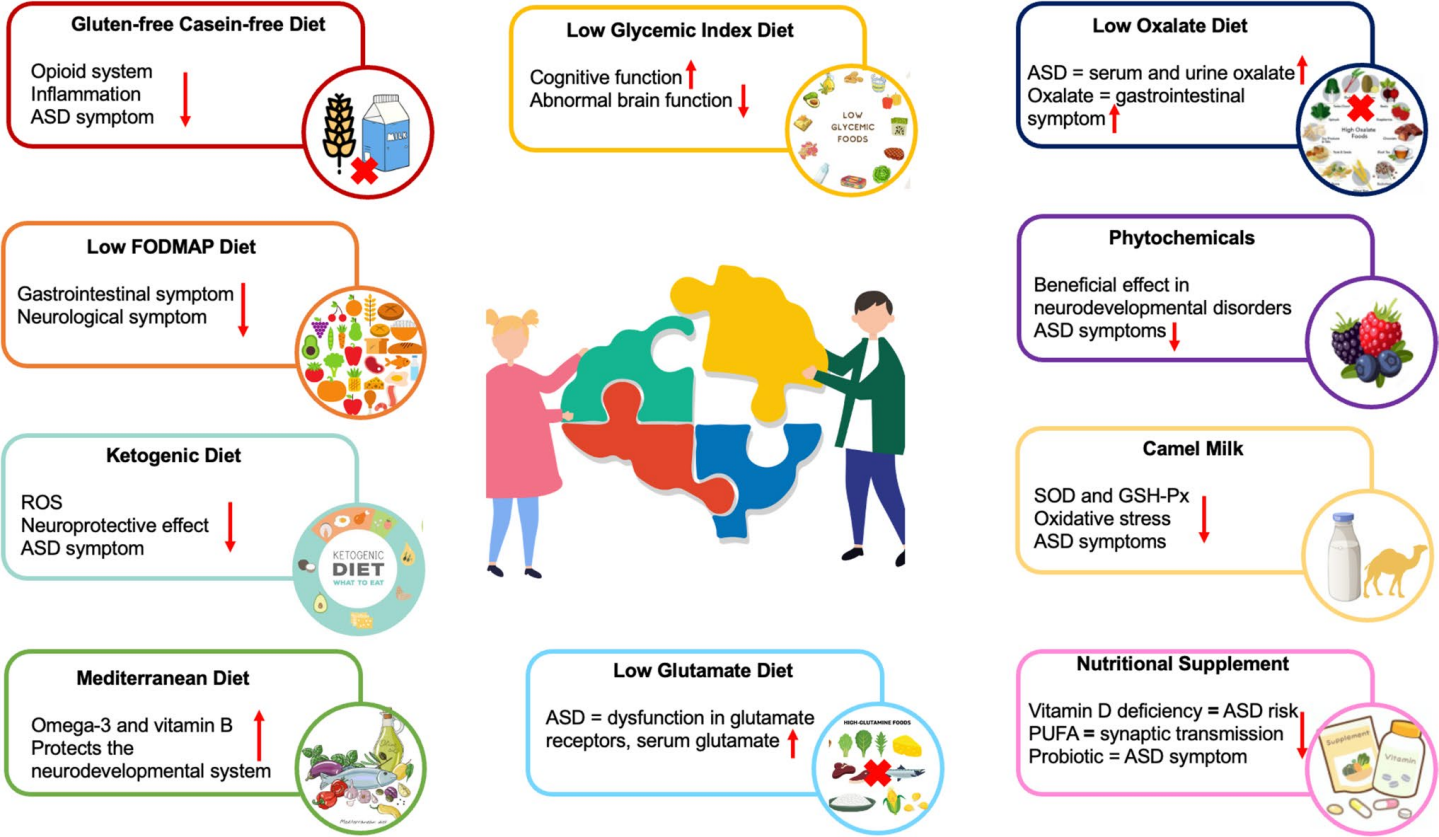
Nutritional approaches in autism spectrum disorder [13, 19, 22, 23]

Nutrition is thought to be one of the environmental factors that influence the symptoms of ASD [21]. Various dietary interventions including GFCF diet, GAPS, FODMAP diet, ketogenic diet, Mediterranean diet, Feingold, Candida body ecology, allergy elimination diets, specific carbohydrate diet, etc.) and supplements (vitamin D, polyunsaturated fatty acids, probiotics and prebiotics, phytochemicals) are used to alleviate these symptoms. Additionally, some nutrients such as camel milk and nutritional supplements such as omega- 3 fatty acids and probiotics may have an effect in alleviating the symptoms of ASD. Some nutritional approaches are shown in Fig. 2 [13, 19, 22, 23]. The gut microbiota of children with ASD differs from the microbiota of children without ASD. Gut microbiota affects gastrointestinal symptoms, behavioral change and mood. Diet affects gastrointestinal functions through its ability to alter the gut microbiome. These nutritional interventions bring both advantages and disadvantages. Restrictive diet may cause food selectivity and nutrient deficiency in individuals with ASD [24].

## Gluten-Free-Casein-Free Diet

Gluten is found in rye, wheat, and barley, while casein proteins are found in milk. Both can have inflammatory effects on the immune system [25]. Gluten and casein induce the expression of lgA and lgG antibodies in children with ASD and may exacerbate its symptoms. Another theory is the “opioid excess” theory, which suggests that gluten and casein peptides are metabolized in the intestine into structurally similar short-chain peptides called gluteomorphins and casomorphins in the behavioral problems seen in these children [16]. The small intestinal mucosa acts as a luminal barrier that prevents such peptides from passing into the bloodstream. In individuals with ASD, intestinal permeability increases due to inflammation and the luminal barrier is altered. The passage of casein, gluten and their metabolites into the bloodstream and central nervous system increases. Increased passage of these metabolites contributes to the development of symptoms of ASD [26].

For all these reasons, some hypotheses suggest that nutritional interventions can improve cognitive function, communication skills, social behaviors, and learning ability [13]. Removal of gluten and casein peptides from the diet in GFCF diet intervention will decrease the possibility of stimulation of the opioid system and alleviate the symptoms of ASD [27].

Casein-free diets involve no milk and dairy products or products containing lactose. In gluten-free diet, gluten-free foods (such as vegetables, fruits, legumes, meat, eggs, and fish) are preferred instead of gluten-containing grains [28, 29]. On the other hand, GFCF diet is a diet protocol that combines casein-free diet and gluten-free diet [29]. Adams et al. [30] applied a gluten-free, soy-free, casein-free diet and vitamin and mineral supplements to individuals with ASD for 12 months. An increase in biomarkers of vitamin A, folic acid, B_2_, B_5_, B_6_, B_12_, EPA, DHA and coenzyme Q10 was detected in the intervention group. Dietary intervention has been reported to be effective in improving nonverbal IQ, ASD symptoms, and nutritional status in most individuals with ASD [30]. In another study, it was observed that applying a normal diet for six months and then a GFCF diet for another six months did not cause any behavioral changes in individuals with ASD [31]. Bavykina et al. [32] applied a GFCF diet to children with ASD for six months. They observed increased levels of specific IgG antibodies against casein in 79.5% of the children. They stated that there was an increase in IgG antigliadin antibodies in 19.3% of children who did not follow a gluten-free diet, and 40–50% of individuals with ASD had gluten intolerance [32]. Piwowarczyk et al. [26] applied a gluten-free diet to children with ASD for six months and reported that the intervention was not effective on their ASD symptoms, behavioral disorders, and intellectual abilities. Matthews and Adams [33] stated that the ketogenic diet and the GFCF diet were the dietary interventions that scored best on ASD symptoms compared to other dietary patterns. ​It is suggested that diets high in gluten and casein may be effective in the pathogenesis of leaky gut syndrome through the gut-brain axis and may synergistically exacerbate dysbiosis as a comorbidity in ASD [33]. However, this study is a retrospective study and is limited to parent-reported data. These results need to be supported by data from larger, prospective studies. In another study, male rats were given diets high in gluten and casein and it was reported that diets rich in gluten and casein may synergistically exacerbate dysbiosis by worsening leaky gut syndrome, by affecting the brain through the gut-brain axis. Both dietary patterns negatively affected the intestinal microbiota and increased intestinal permeability [34]. Another meta-analysis study reported that GFCF dietary intervention may reduce stereotyped behaviors in ASD and elevate the cognition level of children with ASD [35]. In another meta-analysis study, it was determined that GFCF diet had no effect on the basic symptoms, functional level and behavioral problems of children with ASD. Additionally, it was emphasized that dietary intervention may have negative effects on gastrointestinal symptoms [36]. This study provides stronger findings because it is a meta-analysis of randomized controlled trials and the sample size (n = 143) is relatively higher than other studies.

While there are studies showing that the GFCF diet offers effective results on ASD symptoms, there are also studies showing that it has no effect. These conflicting results in the literature indicate that more studies with larger sample sizes should be conducted in this area.

## Ketogenic Diet

The ketogenic diet is a diet model that includes high fat, low carbohydrate, and adequate protein intake. In the classic ketogenic diet, the fat-carbohydrate ratio is 3:1/4:1. Since this ratio makes it difficult to comply with the diet, different ketogenic diet models have been developed such as modified Atkins diet, MCT ketogenic diet and low glycemic index ketogenic diet [37, 38]. Recently, the ketogenic diet has been used in the treatment of various neurological diseases [39–41]. Ketogenic diets elevate the levels of ATP and enzymes related to mitochondrial metabolic pathways and mitochondrial biogenesis. β-hydroxybutyrate, acetoacetate and acetone, known as ketone bodies, are used as the brain’s energy source in case of hunger. Ketone bodies prevent mitochondrial permeability and reduce reactive oxygen species (ROS). Therefore, ketone bodies have a neuroprotective effect on the central nervous system [42]. The ketogenic diet has been suggested to alleviate the symptoms of some neurological diseases by influencing the intestinal microbiota [38]. A systematic review reported that ketogenic diet to neurological patients positively affected the composition of the gut microbiota [43]. Mu et al. [44] applied the ketogenic diet to children with ASD for three months and observed changes in their blood parameters and behavioral problems. At baseline, the children had higher concentrations of galactose intermediates, N-acetylserotonin and trimethylamine N-oxide, and lower concentrations of selenium and 3-hydroxybutyrate than the control group. Circulating ketone bodies and acetylcarnitine and low selenium levels increased following ketogenic diet intervention. Selenium levels were negative correlated with behavior scores [44]. However, this is a pilot study, the sample size is limited and there is no control group. The results need to be confirmed by further studies. In another study, a modified ketogenic gluten-free diet intervention was applied to children with ASD for three months. Significant improvements were observed in the imitation, body use, fear and irritability subscales of the ADOS- 2 and the CARS- 2. It was emphasized that modified gluten-free ketogenic diet intervention with MCT supplementation may be an effective method to improve symptoms in ASD [45]. However, the sample size of this study is limited, and there is no control group. This makes it difficult to determine whether the results are solely attributable to the dietary intervention. More studies are needed to evaluate the effectiveness of the modified ketogenic diet. Another study conducted on BTBR rats with ASD revealed that ketogenic diet intervention changed the mitochondrial function and improved mitochondrial morphology in BTBR rats. Mitochondrial dysfunction and mitochondrial fragmentation play a role in the etiology of ASD [46]. Yu et al. [29] reported that GFCF diet and ketogenic diet significantly improved the basic symptoms of individuals with ASD. Another study revealed that ketogenic diet intervention reduced social deficits, repetitive behaviors, memory impairments, and inflammatory markers such as TNF-α, IL- 1β, and IL- 6 in BTBR rats [47]. In a case report, it was found that the patient’s behavioral problems improved in the first month of a 16-month ketogenic diet intervention in a six-year-old child with ASD symptoms, and 18 FDG PET decreased significantly in 12 months [48]. Epigenetic and synaptic abnormalities are the most important risk factors in ASD. A study conducted on rats revealed that in ASD, there was significantly lower histone acetylation and higher HDAC2 expression in the PFC. Four-week ketogenic diet intervention increased the transcription and histone acetylation of Grin2a and Grin2b in Shank3-deficient rats and ameliorate the decreased NMDAR synaptic function in PFC neurons. The ketogenic diet is reported to be a promising approach for social deficits seen in ASD by regulating gene expression and histone acetylation in the brain [49].

There are studies reporting that the ketogenic diet has a positive effect on ASD symptoms. However, some of the studies in the literature have low reliability due to limited sample size, lack of a control group, or being pilot studies. More studies with larger sample sizes are needed in this area to make a general conclusion.

## Specific Carbohydrate Diet

Specific carbohydrate diet is thought to contribute to the alleviation of microbiota changes, comorbid gastrointestinal symptoms, and behavioral symptoms seen in some individuals with ASD. The specific carbohydrate diet was developed in the 1930 s for the treatment of gluten enteropathy. It has been later used for the treatment of Crohn’s disease, ulcerative colitis, chronic diarrhea and diverticulitis [50]. The aim of this dietary model is to alleviate malabsorption symptoms and prevent the proliferation of pathogenic microflora that results in dysbiosis [50, 51]. While monosaccharide sources such as fruits, some vegetables and honey are used as carbohydrate sources in the diet, complex carbohydrate sources (cereals, potatoes, dairy products, processed foods, and disaccharides including lactose, sucrose, and maltose) are limited [13, 50]. In children with ASD with gastrointestinal problems, implementation of a specific carbohydrate diet protocol has not been evaluated but is an intervention commonly used by many families without clinical guidance. However, further studies are needed to make generalizations on this subject [52]. Barnhill et al. [52] applied a specific carbohydrate diet to a four-year-old boy diagnosed with Fragile X Syndrome and ASD for four months, and they determined that this dietary intervention was well tolerated. Improvements were also observed in gastrointestinal symptoms and behavioral problems after the intervention [52]. Although the specific carbohydrate diet is a widely used dietary intervention, evidence published regarding the effectiveness or safety of this diet in patients with ASD is currently lacking [13].

## Low Glutamate Diet

It is thought that some deficiencies in neurodevelopmental mechanisms may affect the symptoms of individuals with ASD. Neurotransmitters play critical roles in the development of the central and peripheral nervous system. Therefore, neurotransmitter dysfunctions take part in the pathophysiology of ASD [53]. Glutamate is one of the important stimulant neurotransmitters in the human brain. Glutamate receptors are involved in diverse cognitive and neuronal processes such as learning, memory, and synaptic system [54]. Glutamatergic system dysfunction lies at the root of neurotransmitter dysfunction in ASD. Two theories attract attention in glutamatergic system disorder. The first one is hyperglutamate theory, which accepts that glutamate levels are high in the serum and plasma of individuals with ASD, and the second one is the hypoglutamate theory, which states that there is dysfunction in glutamate receptors in ASD and glutamatergic agonists may have a positive effect on some of ASD symptoms [55].

Carvalho et al. [56] showed in their study that total N-acetylaspartate (tNAA) levels lowered in individuals with ASD and Gamma aminobutyric acid (GABA) was associated with communication skills and developmental scores, which are the main symptoms of ASD. Naaijen et al. [57] reported that N-acetylaspartate (NAA) and glutamate play a role in striatal volume in individuals with ASD, and low NAA in ASD indicates decreased neuronal integrity and impaired neuronal functioning. Siegel-Ramsay et al. [58] conducted a study of adults with ASD and found that there was a significant negative correlation between increased glutamine concentrations and decreased functional connectivity in the insular, limbic and parietal regions of the dorsal anterior cingulate cortex (dACC). A study conducted on children with ASD in Egypt reported that children with ASD had higher levels of serum glutamate and this parameter may help diagnosing ASD in the early period [59]. In a study conducted on rats with ASD, it was observed that the NAA/total creatine (tCr) ratio, glutamate/GABA ratio and tCr level in the prefrontal cortex were substantially associated with the lack of social behavior in rats [60]. A study investigating d-aspartate metabolism in the brain and serum of rats with ASD that the levels of mRNA of d-aspartate oxidase, which encodes the enzyme, responsible for d-aspartate catabolism, significantly lowered [61].

A low glutamate diet acts on N-methyl-D-aspartate receptors. It reduces the negative effects of dietary amino acids such as aspartate and glutamate, which cause excitotoxicity in the long term, and can be used in the treatment of some diseases. In a low glutamate diet, the free or esterified form of glutamate is eliminated from the diet. Foods such as soy sauce, old cheese and mushrooms can be given as examples of foods containing free glutamate. The free form of aspartate is also limited in this dietary model because it activates N-methyl-D-aspartate receptors. Also, it is recommended for individuals with ASD to consume a diet rich in antioxidants to protect them from oxidative stress caused by glutamate excitotoxicity [62]. There has been no study investigating the efficiency of a low glutamate diet in ASD in the literature. The effect of a low glutamate diet on high glutamate levels in the serum and plasma of individuals with ASD is unknown. More advanced clinical studies are needed in this field.

## Low FODMAP Diet

Fermentable oligosaccharides, disaccharides, monosaccharides, and polyols (FODMAP) are a heterogeneous group of poorly absorbed short-chain carbohydrates that can be fermented in the small intestine or large intestine. FODMAPs include lactose (milk and dairy products), fructose (fruits and high fructose corn syrup), fructans (vegetables, fruits, and grains), galactans (legumes and vegetables) and sugar alcohols (fruits, vegetables polyols, and sorbitol) [63]. The low FODMAP diet is a dietary model that has long been used to improve symptoms such as constipation, diarrhea, bloating, and abdominal pain. It is thought that gastrointestinal symptoms observed in ASD are associated with carbohydrate metabolism. In children, a low FODMAP diet may alleviate symptoms of ASD, including irritable bowel syndrome [51, 64]. Nogay et al. [65] applied a low FODMAP diet to children with ASD for two weeks. They observed that the individuals’ gastrointestinal syndromes alleviated significantly and there was no significant difference in behavioral problems [59]. In another case report, the ketogenic diet and the low FODMAP diet were applied to a 17-year-old girl with ASD. It was observed that the patient with ASD tolerated the low-FODMAP diet better than the ketogenic diet, and the low-FODMAP diet significantly improved neurological, gastrointestinal and metabolic symptoms [66].

The number of studies in the literature on the effectiveness of the FODMAP diet on ASD symptoms is quite limited. Longer-term studies with larger sample sizes are needed to make generalizations.

## Mediterranean Diet

The Mediterranean Diet contains high amounts of vegetables, fruits, olive oil, legumes, and oilseeds, moderate amounts of fish, dairy products, and wine, and small amounts of red meat and saturated fat [67]. This diet ensures adequate intake of n- 3 fatty acids and B vitamins, which have protective effects on neurodevelopment in individuals with depression or ASD [64]. There has been no study investigating the relationship between the Mediterranean diet and ASD in the literature. However, some studies have examined the effect of the Mediterranean diet on the neurodevelopmental system and its effect on attention deficit and hyperactivity disorder (ADHD).

In a study conducted on 1471 mother–child pairs in China, it was observed that the mother’s adherence to the Mediterranean diet during pregnancy was associated with lower neurodevelopmental risk in children [68]. In another study, high adherence to the Mediterranean diet in the first trimester of pregnancy was associated with higher communication skills in 6-month-old infants [69]. Ríos-Hernández et al. [70] showed that adherence to the Mediterranean diet was lower in children with ADHD. In another study, the Mediterranean diet and n- 3 fatty acid supplements were given to the individuals with ADHD. It was observed that n- 3 fatty acid was associated with less pronounced impulsive behavior. Applying the Mediterranean diet may improve the results, but further studies are needed [71].

The number of studies on the effect of the Mediterranean diet on ASD symptoms is limited. In addition, studies are mostly on compliance with the Mediterranean diet. Further studies should be conducted to investigate the effectiveness of the Mediterranean diet on ASD.

## Low Glycemic Index Diet

The glycemic index is defined as a scale that ranks the effect of a carbohydrate-containing food on blood glucose compared to a reference food (glucose). If the glycemic index of a food is ≤ 55, it is grouped as low glycemic index, and ≥ 70 as high glycemic index [72]. Low glycemic index diet is used for body weight regulation and glycemia control in individuals with diabetes mellitus. In addition, this dietary pattern has an effect on brain functions. For this reason, it is used to improve cognitive functions (such as attention and memory) in individuals or to modulate abnormal brain functions in ASD, epilepsy and other neurological disorders [73].

Currais et al. [74] applied low-and high-glycemic index diets to BTBR rats. They reported that while the low glycemic index diet lowered levels of advanced glycation end products (AGEs) and other inflammatory markers in plasma and brain, the high glycemic index diet affected many parameters that were thought to lead to ASD.

There are very few studies in the literature investigating the effect of low glycemic index on ASD. This makes it very difficult to draw any conclusions about the effect of low glycemic index diets on ASD. More studies with larger samples are needed in this area.

## Other Dietary Interventions, Nutrients and Food Additives

In individuals with ASD, some substances such as oxalates may be associated with gastrointestinal dysfunction, neurological development, and disruption of the central nervous system structure and functionality in the nervous system [16, 75]. The high level of oxalate in the blood serum and urine of individuals with ASD suggests that oxalate may be effective in in neurobiological mechanisms of ASD [16, 22]. ADI (acceptable daily intake) of oxalates for an adult is 250 mg/g. In the Western diet, daily dietary intake of oxalate can reach up to 1000 mg/g. For individuals with ASD, it is recommended to restrict foods with high oxalate content and daily dietary oxalate intake to 40–50 mg. Since limiting foods such as figs, green apples, kiwis, beets, spinach, and tangerines, which are rich in oxalates, may cause micronutrient deficiency, vitamins A and E, magnesium, calcium and zinc supplements are recommended for individuals with ASD who follow a low oxalate diet [22]. Although dietary models that restrict oxalate have been developed for ASD, there is no evidence-based study yet on the effectiveness of this diet in ASD [19]

It has been said for many years that yeasts play a role in the behavior and learning problems of children with ASD. Excessive antibiotic exposure causes intestinal dysbiosis and leads to the proliferation of C. albicans yeasts in the intestine. Candida can cause ASD by allowing toxic substances to pass into the bloodstream and affecting the brain functions and immune system. However, this condition is not seen in all children with ASD. For this reason, it is not correct to conclude that Candida causes ASD. Candida species proliferate excessively in the intestines of individuals with ASD [76]. Iovene et al. [77] found in their study that 57.5% of individuals with ASD had the aggressive form of Candida species. They emphasized that carbohydrate consumption was also effective in the increase of Candida species in children with ASD. In a study including children with ASD, C. albicans, C. dubliniensis and C. tropicalis species were detected in the stools of the majority of children with ASD who consumed a carbohydrate-free diet. C. parapsilosis and C. albicans were found in the stools of children with ASD fed a carbohydrate-containing diet, and it was reported that carbohydrate consumption may differ the Candida species in the stools of children with ASD [78]. A study conducted on rats indicated that carbohydrates and sugar alcohols caused growth of C. Albicans [79]. The anti-candida diet aims mainly to reduce carbohydrates. This diet is a dietary pattern that limits highly refined carbohydrates, added sugars (including sugar, honey, jam), dairy products, and cured and red meats. However, there is no study on whether this dietary model can alleviate symptoms in individuals with autism spectrum disorder [76].

The Feingold diet does not include foods containing grapes, almonds, oranges, apples, peaches, strawberries, pickled cucumbers, artificial food colorings, sweeteners, flavorings and preservatives as well as foods containing BHA, TBHQ, BHT and salicylates [23, 33]. The Feingold diet is one of the oldest diet models used in the management of ADHD [80]. This is an elimination diet used for children with ASD, as well [23]. Abdallah et al. [81] applied the Feingold diet to children with ASD and gave them a language education. Insignificant improvements in receptive language skills and language training performance were seen after the intervention [81]. In a study comparing 13 dietary interventions in ASD, it was found that the Feingold diet did not have any side effect and ranked first in six areas related to behavior, including aggression, sensory sensitivity, hyperactivity, irritability, falling asleep and staying asleep, anxiety, attention, and sleep. In terms of cognition, it was in the top three when compared to other dietary interventions [33].

The basic principles of the GAPS diet are the formation of an optimal microbiota for the proper functioning of the body [82]. This dietary pattern is a nutritional model that aims to improve the damaged intestinal microbiota and is used in ASD, ADHD, depression, dyspraxia, dyslexia, schizophrenia, obsessive compulsive disorder, epilepsy, bipolar disorder, and many other conditions that affect brain functions [83]. GAPS diet frequently includes high-density, easily digestible foods with high nutritional values. All foods that are difficult to digest and negatively affect the microbiota are eliminated from this diet. All food is prepared in house with fresh natural ingredients. Fermented foods are widely used in this nutritional model [82], in which grains (wheat, barley, oats, corn, rice), grain products (bread, pasta, etc.), starchy vegetables, milk, sugar, artificial sweeteners, and packaged and processed products are prohibited and home-cooked meat, fish, poultry, juices and soups of vegetables with low starch content, homemade yoghurt, kefir, ginger and chamomile tea are recommended. As symptoms alleviate, organic eggs, ghee oil, avocado, cold-pressed olive oil, freshly squeezed fruit juices, oilseeds and fruit purees are added to the diet [83].

Ābele et al. [84] applied a specific carbohydrate diet/GAPS diet to children with ASD and those having an unclear diagnosis, and n- 3 essential fatty acids, probiotics, ascorbyl palmitate vitamin C and vitamin D were supplemented. An important decrease was observed in the gastrointestinal system symptoms of the intervention and control groups. Specific carbohydrate diet/GAPS and dietary supplements may be a secure method to improve some symptoms of ASD in children [84]. However, there is no enough evidence in the literature for the GAPS diet and further randomized controlled studies are needed.

The number of studies investigating the effectiveness of other dietary interventions such as anti-candida, Feingold, GAPS diet on ASD symptoms is quite limited. More comprehensive studies are needed to investigate the long-term effects of these diets and interventions and to what extent they are useful in clinical practice.

While the lactose content of camel milk is lower than the content of cow milk, its MCT, vitamin (A, E C) and mineral (Ca, Fe, Mg, Cu, Zn, K) content is higher [19, 85, 86]. Low plasma glutathione (GSH) and cysteine levels are associated with ASD. It has been shown that camel milk alleviates the clinical symptoms of ASD by elevating glutathione peroxidase (GSH-Px) and superoxide dismutase levels [87]. In addition, it may have beneficial effects by reducing oxidative stress, which is considered one of the etiologies of ASD [86].

In the meta-analysis study conducted by Kandeel et al. [88], it was reported that camel milk had a positive effect on the social behavior of children with ASD, but did not cause a statistically significant difference in CARS scores. This study offers stronger results because it is a meta-analysis of randomized controlled trials and the sample size is higher than other studies. In another study, children with ASD were supplemented with raw camel milk, cooked camel milk, and placebo (cow’s milk) for two weeks. Both raw and cooked camel milk reduced neuroinflammation in these children and increased their Social Responsiveness Scale (SRS) score and gastrointestinal symptoms [89]. Hamzawy et al. [90] stated that camel milk supplementation in pregnant female rats regulated inflammatory and apoptotic pathways and may be a potential beneficial agent for ASD.

Food additives are substances added to foods to provide taste, appearance and other desirable properties [91]. One of the most current debates in this field is the negative impact of artificial food dyes on children’s behaviors [92]. One concern the researchers have about the adverse neurobehavioral effects of food additives is that artificial food dyes are among the etiological factors in childhood hyperactivity. In a study conducted on individuals with ADHD, subjects in the intervention group consumed chocolate chip cookies containing artificial food dyes three days a week for two weeks. It was observed that artificial food dyes caused an increase in inattention symptoms in these individuals [93]. It has not been fully proven that food dyes cause ASD. However, high doses of food additives may cause pharmacologically an adverse effect in a small percentage of children with ASD [92].

## Dietary Supplements

In some children with ASD, inadequate nutritional intake, vitamin-mineral and trace element deficiencies, low vitamin levels in the body and brain, and the formation of antibodies against vitamin carriers in the blood–brain barrier are observed. Gastrointestinal inflammation seen in ASD increases the risk of nutrient deficiency [94]. Esteban-Figuerola et al. [95] reported that individuals with ASD consume lower amounts of vitamin D, calcium and dairy products and higher amounts of fruits, vegetables, protein, selenium, phosphorus, riboflavin, thiamine, and vitamin B12. Nutritional interventions to alleviate symptoms of ASD include vitamins, minerals, polyunsaturated fatty acids and probiotic supplements in addition to dietary approaches [23].

## Vitamin D

Vitamin D is a fat-soluble steroid hormone that is essential for the human body. Vitamin D3 is synthesized in the skin as a result of the reaction of 7-dehydrocholesterol with ultraviolet B (UVB) rays [96, 97]. In recent years, it has been frequently discussed that vitamin D deficiency is one of the environmental factors that cause ASD. Although the underlying mechanisms between vitamin D and ASD are not clearly clarified, there is some biological evidence showing the relationship between them [96]. Vitamin D plays an important role in neurodevelopment. Neurotransmission, neural cell proliferation, immune functions and oxidative stress are among the main mechanisms mediated by vitamin D in the central nervous system [98]. Vitamin D has important effects on brain development and functions, such as proliferation, immune modulation, and regulation of synaptic plasticity. Considering all these factors, vitamin D deficiency during pregnancy and early childhood can negatively affect brain development of children and cause many neuropsychological disorder, especially ASD [96].

Petruzzelli et al. [99] found that serum 25-hydroxylvitamin D (25(OH)D) concentration was lower in children with ASD than in those without ASD. They stated that vitamin D deficiency may be a risk factor for ASD [99]. In a cohort study including families having children with ASD, a strong correlation was observed between one of the VDR (Vitamin D receptor) polymorphisms and hyperactivity behavior in children with ASD. It was reported that the same VDR polymorphism in mothers without ASD may increase the risk of giving birth to a child with ASD [100]. Another meta-analysis study reported that vitamin D supplementation in children with ASD reduced SRS and CARS- 2 scores and alleviated the symptoms of ASD [101]. This study, being a meta-analysis and including randomized controlled trials, makes the findings stronger compared to other studies. Moradi et al. [102] applied vitamin D supplementation and perceptual motor exercises to children with ASD. They observed that the combination of vitamin D3 supplementation and perceptual-motor exercises led to a significant decrease in stereotyped behaviors [102]. In another study where vitamin D (300 IU/kg/g) supplementation was applied to children with ASD for 15 weeks, it was observed that 86% of children with ASD had vitamin D deficiency. Vitamin D supplementation reduced CARS and Autism treatment Checklist (ATEC) scores, but did not change IL- 6 and ABC scores, and may have a beneficial effect on ASD symptoms [103].

There are some studies in the literature showing that serum vitamin D levels are low in individuals with ASD and that supplementation may improve symptoms. However, larger sample sizes and longer-term studies should be conducted to make generalization from this area.

## Polyunsaturated fatty acids

Polyunsaturated fatty acids (PUFAs) have at least two carbon–carbon double bonds in their carboxylic chains. PUFAs are classified as n- 3, n- 6, n- 9 according to their distance from the methyl group located at the end of the molecule in the first double bond [104]. The most abundant PUFAs in brain tissue are n- 3 and n- 6. Both groups of fatty acids play an important role in the development of the central nervous system. n- 3 and n- 6 fatty acids are involved in the regulation of brain processes such as neurite outgrowth, neurogenesis and synaptogenesis [105]. EPA and DHA act on the synaptic membrane and signal transmission [104]. Recently, the effect of PUFAs on the cognition and behavioral disorders of individuals with ASD has been frequently investigated. The benefits of PUFAs on these individuals are explained as increasing the efficiency of synaptic transmission and modulating neurotransmitter release [105]. Doaei et al. [106] reported that daily 1000 mg omega- 3 fatty acid supplementation to children with ASD alleviated ASD-related symptoms, including social communication. In a study conducted in New Zealand, where children with ASD were supplemented with vitamin D (2000 IU/day) and n- 3 (722 mg/day DHA) for 12 months, it was observed that their irritability and hyperactivity symptoms considerably alleviated [107]. In another study, premature infants (18–38 months) were given n- 3, n- 6 and n- 9 fatty acid supplements for 3 months. Fatty acid supplementation improved socio-emotional parameters, especially in children suffering from symptoms common in ASD [108]. A meta-analysis study investigating the effect of n- 3 fatty acid supplementation on the symptoms of children with ASD reported that n- 3 and n- 6 supplementation alleviated ASD symptoms according to the ABC, but had no effect on irritability, inappropriate speech, and hyperactivity, which are subgroups of this scale [109]. However, the dosage of n- 3 fatty acids varies significantly based on various important factors such as duration of supplementation, severity of ASD, and type of fatty acid supplemented. Large-scale clinical studies are needed before definitive conclusions can be drawn.

## Probiotic and Prebiotic

Probiotics are live microorganisms that provide health benefits to the host. Prebiotics, on the other hand, are substrates that are selectively used by host microorganisms and have a beneficial effect on health [110]. Although probiotics have therapeutic properties in gastrointestinal system diseases, they have also recently been used in the treatment of psychological symptoms such as depression and anxiety. Gut microbiota is involved in modulating the gut-brain axis. Dysbiosis causes psychological symptoms along with gastrointestinal system symptoms [111]. There is evidence that probiotics and prebiotics alleviate gastrointestinal tract symptoms and behavioral problems in ASD [112]. Grimaldi et al. [113] applied prebiotic supplementation to children with ASD for 6 weeks and observed that prebiotic supplementation provided an important reduction in gastrointestinal complaints such as abdominal pain and bowel movements. Another study conducted on children with ASD reported that probiotic supplementation increased *Lactobacillus* species in the intestines and alleviated gastrointestinal symptoms, while no side effects were observed [114]. In their study, Li et al. [115] determined that probiotic supplementation to individuals with ASD for 3 months provided a significant decrease in ATEC score. The relative abundance of *Bifidobacterium*, *Lactobacillus*, *Coprobacillus*, *Ruminococcus*, *Prevotella*, and *Blautia* was significantly higher after supplementation, and the abundance of *Shigella* and *Clostridium* was significantly lower, meaning that probiotic supplementation regulated the intestinal flora [115]. In another study, bovine colostrum and probiotic supplements with high prebiotic content were given to children with ASD aged between 2 and 11 months, and it was stated that the supplement reduced the frequency of gastrointestinal symptoms and abnormal behaviors [116]. In a study including probiotic and fructooligosaccharide supplementation to children with ASD, it was reported that the intervention was associated with ASD symptoms, including dopamine metabolism disorder and hyper-serotonergic state, and modulated the gut microbiota [117]. In addition to the mentioned dietary supplements, it has been reported that micronutrients such as vitamin B6, vitamin B12, vitamin C, vitamin A, folate and magnesium may be associated with ASD [13]. Although many dietary interventions have been investigated for the management of ASD, the effect of dietary interventions or supplements on ASD is unclear. It would not be correct to make a definitive recommendation for a specific nutritional intervention.

## Other Nutrients and Components

### Phytochemicals

Dietary phytochemicals such as carotenoids, isoprenoids, polyphenols, phytosterols and saponins are associated with many diseases such as obesity, cancer and cardiovascular diseases [118]. Additionally, the potential beneficial effect of phytochemicals in neurodevelopmental disorders such as ASD has recently attracted attention [119]. Sulforaphane increases detoxification, regulates cytoprotective enzymes, and removes toxic substances and free radicals from the body. Twelve weeks of sulforaphane supplementation to children with ASD and related neurodevelopmental disorders significantly improved ABC and SRS scale scores, and urinary metabolites (amino acid/gut microbiome, neurotransmitters, oxidative stress, sphingomyelin and hormones) related to changes in symptoms can be used to determine pathways playing an role in action mechanism of interventions aimed at alleviate ASD symptoms [120]. In another study, significant improvements were observed in the ABC scale score of 36-week sulforaphane supplementation in children with ASD compared to the placebo group [121]. Lynch et al. [122] reported that sulforaphane supplementation to individuals with ASD for 3 years could alleviate the symptoms of ASD.

Curcumin is the bioactive compound of turmeric and has a neuroprotective effect [123]. It has been shown that 20 mg/kg/day curcumin supplementation to BTBR rats reduces repetitive behaviors and improves ASD-related symptoms and cognitive impairment [124]. Jayaprakash et al. [125] reported that curcumin supplementation strengthened α7-nAChRs in central nervous system neurons, reduced oxidative stress, and improved ASD symptoms in BTBR rats.

Lutein is a fat-soluble xanthophyll carotenoid that is found in the brain of newborns and plays a significant role in neurodevelopment in their first years of life. It may be effective in alleviating the symptoms of ASD. In a study, lutein supplementation was administered to rats showing ASD-like symptoms caused by valproic acid exposure for 14 days. As a result of the intervention, reductions were observed in ASD symptoms such as social memory deficit, repetitive behaviors, and biomarkers of oxidative stress and apoptosis in the hippocampus [126]. In another study in which beta-carotene supplementation was performed on BTBR rats, it was reported that oral beta-carotene supplementation to infants of families prone to ASD after birth may be effective in preventing and/or alleviating ASD symptoms [127].

Naringenin is a flavonoid compound that is abundant in grapefruit, orange and tomato peels, has antioxidant properties [86] and is a potent neuroprotective through its direct or indirect effect on various pathological pathways. In a study in which naringenin supplementation was administered for 29 days to rats exhibiting ASD-like behavior due to propanoic acid exposure, naringenin showed beneficial effects by alleviating neuropsychopathology of ASD [128].

Quercetin is a polyphenol belonging to the flavonoid group found in vegetables and fruits and significantly reduces ROS, neuroinflammation, anxiety risk, and stress-related mood swings [129]. Owing to its antioxidant properties Quercetin can reduce oxidative stress associated with ASD. It was observed in a study that 40 mg/kg/g quercetin supplementation to rats could reduce ASD symptoms and oxidative and apoptotic damages [130]. Supplementation of 50 mg/kg/g quercetin to rats showing ASD-like behavior with the application of valproic acid showed a neuroprotective effect [131].

Resveratrol is a polyphenolic stilbenoid found in grapes, pine nuts, peanuts and red wine and shows antioxidant, anti-inflammatory and neuroprotective properties [132, 133]. It is known that individuals with ASD have a high level of oxidative stress and an impaired immune system. Therefore, resveratrol may contribute to ASD-related mechanisms [133]. It was shown that 200 mg/g resveratrol supplementation to children with ASD for 3 months significantly reduced the ABC score, and resveratrol supplementation modulated behavioral changes and immune system-related markers [134]. Hendouei et al. [135] reported that children with ASD received 250 mg resveratrol supplements twice a day for 10 weeks and this supplementation did not have any effect on their irritable behaviors, but could recover hyperactivity and noncompliance. Rats experiencing ASD-like symptoms were supplemented with propanoic acid and resveratrol (5, 10, and 15 mg/kg) for 28 days. Resveratrol alleviated these symptoms by suppressing mitochondrial dysfunction, oxidative-nitrosative stress, TNF-α and MMP- 9 expression in rats. Resveratrol can be used to manage neurobehavioral and biochemical disorders in ASD [136].

Bioactive compounds such as sulforaphane, curcumin, lutein, resveratrol, quercetin and naringenin may have potential positive effects on ASD. There are also studies in the literature reporting that these compounds do not show any effect. In addition, there is not yet sufficient information on the long-term effects of phytochemicals. Large-scale and long-term studies should be conducted to better understand the role of phytochemicals in ASD.

Major clinical studies conducted to evaluate the effects of various dietary interventions and supplements on the symptoms of ASD are summarized in Table 1.

**Table 1:** Dietary interventions and supplements in autism spectrum disorder: animal and clinical studies

Sample	Intervention	Outcomes	Reference
BTBR and C57BL/6 J (B6) rat (control group)	Group 1: standard diet B6 rats Group 2: KD B6 rat Group 3: standard diet BTBR rat Group 4: KD BTBR rat KD: 6.3:1	It was observed that KD caused lower movement threshold and higher excitation/inhibition in BTBR rats compared to C57BL/6 J (B6) controls. KD increased cortical excitability in rats	[137]
3–8 year old child with ASD (n = 45)	Group 1: modified Atkins diet (KD) Group 2: GFCF Group 3: control Intervention time: 6 months	Both diet groups showed significant improvement in ATEC and CARS scores compared to the control group. KD provided better results in terms of cognition and sociability than the GFCF diet group	[138]
Juvenile male C57BL/6 (B6) and BTBR rat	BTBR rat: KD C57BL/6 (B6) rat: standard diet Response time: 10–14 days	KD ameliorated some neurological symptoms associated with ASD by reducing the total intestinal microbial count and compositional restructuring in BTBR rats	[139]
Children with ASD aged 4–16 (n = 80)	Intervention group: gluten-free diet Control group: standard diet Intervention time: 6 weeks	Gluten-free diet intervention resulted in significant reductions in gastrointestinal symptoms and behavioral problems	[140]
3–5 year old child with ASD (n = 14)	After 6 weeks of GCFC intervention 12 weeks gluten, casein, gluten + casein and placebo groups	The intervention showed no significant effects on physiological functioning, behavioral problems, and ASD symptoms	[141]
2–12 year old child with ASD (n = 45)	Group 1: cooked camel milk, 500 mL Group 2: raw camel milk, 500 mL Group 3: placebo	Serum TARC levels decreased significantly in group 1 and group 2. Significant improvements were seen in the CARS score only in the raw camel milk supplement group	[142]
Child with ASD (n = 83)	Vitamin D3 (300 IU/kg/day, max: 5000 IU) for 3 months	Vitamin D3 supplementation resulted in significant improvement in 80.72% of children with ASD on sections of the CARS and ABC subscales measuring behavior, stereotypy, eye contact, and attention span	[143]
7–18 year old child with ASD (n = 41)	1 g omega- 3 fatty acid supplement twice daily, 12 weeks	Significant improvements were seen in all subscales of the SRS and the CBCL scale	[144]
2.5–8 year old child with ASD (n = 73)	Group 1: Vitamin D (2000 IU/day) Group 2: omega- 3 LCPUFA (722 mg/day docosahexaenoic acid) Group 3: Vitamin D (2000 IU/day) + omega- 3 LCPUFA (722 mg/day docosahexaenoic acid) Group 4: placebo Intervention period: 12 months	The intervention modulated the inflammatory status (IL- 1β) in children with ASD and improvements in SRS scores were observed	[145]
4–9 year old child with ASD (n = 13)	Prebiotic (partially hydrolyzed guar gum), 6 g/day	Partially hydrolyzed guar gum supplementation to children with ASD who experienced constipation alleviated the symptoms of constipation and intestinal dysbiosis. A significant decrease was observed in serum inflammatory cytokines and the behavioral irritability subscale of ABC	[146]
5–9 year old child with ASD (n = 30)	Probiotic supplement (*Lactobacillus acidophilus*, *Lactobacillus rhamnosus* and *Bifidobacteria longum*), powder form, 5 g/day, 3 months	Improvements were seen in ATEC and gastrointestinal symptoms (6-GSI)	[147]
18–72 month old child with ASD	Group 1: GI probiotic (n = 9) Group 2: NGI probiotic (n = 22) Group 3: GI placebo (n = 8) Group 4: NGI placebo (n = 24) Intervention time: 6 months	There was a significant decrease in ADOS-CSS scores in Group 2 compared to the placebo group. Greater improvements in some GI symptoms, adaptive functioning, and sensory profiles were seen in group 1 compared to group 3	[148]
Male Sprague–Dawley rat	With the application of 1 M propanoic acid, rats were caused to show ASD-like symptoms curcumin supplement (50, 100 and 200 mg/kg), 4 weeks	Curcumin supplementation improved the core of the autistic phenotype and associated symptoms by suppressing mitochondrial dysfunction, oxidative-nitrosative stress, TNF-α and MMP- 9 in rats	[149]
4–12 year old child with ASD (n = 60)	Sulforaphane daily 50 μmol (≤ 45 kg) 100 μmol (> 45 kg)	Sulforaphane supplementation improved symptoms of irritability and hyperactivity in children with ASD	[150]
4–10 year old child with ASD (n = 40)	Product supplement containing lutein and quercetin, 26 weeks	After the intervention, a decrease in serum inflammatory cytokine levels (IL- 6 and TNF) and an improvement in the general symptoms of ASD were observed	[151]
Female pregnant wistar rat	Rats were divided into 4 groups: Control group (n = 7), RSV group (n = 7), VPA group (n = 12–19) and VPA + RSV group (n = 9–10) Response time: 13 days Prenatal VPA application to rats was made to exhibit ASD-like behavior	Prenatal administration of RSV prevented VPA-induced social impairments	[152]
Children with ASD aged 2–18 (n = 247 with ASD, n = 267 healthy controls)	In a case–control study, the prevalence and types of gastrointestinal symptoms, frequency of food selectivity, and mealtime difficulties were investigated	A gastrointestinal severity index questionnaire was administered. A higher prevalence of gastrointestinal symptoms was observed in girls with ASD than in boys. The incidence of mealtime difficulties (64.3%) and food selectivity (69.1%) is high. Behavioral traits had weak but significant correlations with gastrointestinal symptom frequency	[153]

**Abbreviations** ASD: Autism spectrum disorder, KD: Ketogenic diet, GFCF: gluten-free-casein-free diet, ATEC: Autism Treatment Evaluation Test, CARS: Childhood Autism Rating Scale, ABC: Abnormal behavior checklist, 6-GSI: Gastrointestinal Severity Index, GI: Gastrointestinal symptoms, NGI: Non-gastrointestinal, ADOS-CSS: Total Autism Diagnostic Observation Schedule—Calibrated Severity Score, TNF-α: Tumor necrosis factor-α, MMP- 9: Matrix metalloproteinases, IL- 6: Interleukin- 6, SRS: Social Responsiveness Scale, CBCL: Social and Attention Problems syndrome of Child Behavior Checklist, IL- 1β: Interleukin- 1β, TARC: Thymus and activation-regulated chemokine, RSV: Resveratrol, VPA: Valproic acid, LCPUFA: Long-chain polyunsaturated fatty acid

## Conclusion and Recommendations

There is no nutritional intervention that has a definitive and clear effect in managing ASD. Although nutritional interventions have shown a positive effect on symptoms in some individuals with ASD, the literature does not provide a definitive result due to reasons such as limited sample size in the studies conducted. Since the symptoms of ASD are specific to the individual, nutritional interventions differ for each individual. Nutrition of individuals with ASD should focus on reducing behavioral symptoms and eliminating troubles. In order to create the ideal diet model that can be used for its management, to determine the effect of nutrition on autism behaviors and to make correct nutrition plans, prospective epidemiological studies with large samples and clinical studies with in vivo, in vitro, animals and humans are needed.

## Future Perspective

In light of these reviews, the efficiency of dietary interventions and supplements in ASD appears to be a strong and evolving topic of debate. No clear conclusion can be made as to which nutritional intervention is more appropriate for alleviating ASD symptoms. Future research initiatives in dietary patterns need to focus on increasing the bioavailability of nutrients. Larger-scale and longer-term clinical studies are needed to target and fill existing gaps in the relevant literature. Substantial evidence is needed to reveal clinical practices and protective effects on health, and to evaluate potential risks from nutrition in individuals with ASD. However, current scientific evidence is still insufficient to create a consensus regarding the use of foods and dietary patterns in reducing and managing ASD symptoms.

Since the nutritional patterns and consequences of children with autism are not yet fully understood and clarified, it is important to conduct further studies that will include the process of shaping the eating habits of these individuals, their eating behaviors, the effects of the applied diet on their daily eating habits and the formation of food selectivity. In addition, it is needed to conduct comprehensive, large-scale, longer-term randomized controlled in vivo, in vitro, human and animal clinical studies in order to identify, evaluate, and improve the effectiveness of dietary interventions and supplements in ASD, to ensure safety, and to provide a better understanding of potential health effects.

## Key References

- Olivito I, Avolio E, Minervini D, Soda T, Rocca C, Angelone T, et al. Ketogenic diet ameliorates autism spectrum disorders-like behaviors via reduced inflammatory factors and microbiota remodeling in BTBR T+Itpr3tf/J mice. Exp Neurol. 2023;366:114,432.10.1016/j.expneurol.2023.114432.
  - This article investigated the effects of ketogenic diet on rats. It was reported that ketogenic diet reduces social deficits, repetitive behaviors, memory impairments and inflammatory markers such as TNF-α, IL- 1β and IL- 6 and may be a therapeutic approach in autism spectrum disorder.
- Kandeel M, Morsy MA, Al Khodair KM, Alhojaily S. Meta-analysis of the efficacy of camel milk consumption for improving autism symptoms in children in randomized clinical trials. *Open Vet J*. 2024;14(9):2441–52.10.5455/OVJ.2024.v14.i9.33.
  - This article is a meta-analysis study examining the effect of camel milk on autism spectrum disorder. It has been reported that camel milk may have positive effects on the social behavior of children with autism spectrum disorder.
- Viana CE, Bortolotto VC, Araujo SM, Dahleh MMM, Machado FR, de Souza Pereira A, et al. Lutein-loaded nanoparticles reverse oxidative stress, apoptosis, and autism spectrum disorder-like behaviors induced by prenatal valproic acid exposure in female rats. Neurotoxicology. 2023;94:223–34. 10.1016/j.neuro.2022.12.006.
  - This article is an animal study examining the effect of lutein on ASD symptoms. Lutein-loaded nanoparticles were reported to reduce ASD symptoms such as social memory deficit, repetitive behaviors, and biomarkers of oxidative stress and apoptosis in the hippocampus after valproic acid exposure in female rats.

## Supplementary Information

Below is the link to the electronic supplementary material.

Supplementary file1 (PDF 200 KB)

## Data Availability

All data needed to evaluate the conclusions in this article are included in the article. Additional data related to this article may be requested from the authors.

## Abbreviations

ABS: Abnormal behavior checklist
ADI: Acceptable daily intake
ADOS- 2: Autism diagnostic observation schedule
AGEs: Glycation end products
ASD: Autism spectrum disorder
ATEC: Autism treatment evaluation test
ATP: Adenosine triphosphate
BHA: Butylated hydroxyanisole
BHT: Butylated hydroxytoluene
CARS- 2: Childhood autism rating scale
dACC: Dorsal anterior cingulate cortex
DHA: Docosahexaenoic acid
DSM- 5: Diagnostic and Statistical Manual of Mental Disorders fifth edition
EPA: Eicosapentaenoic acid
FODMAP: Fermented soluble oligosaccharides disaccharides monosaccharides and polyols
GABA: Gamma aminobutyric acid
GARS- 2: Gilliam autism rating scale
GFCF: Gluten-free-casein-free
GSH: Glutathione
GSH-Px: Glutathione peroxidase
HDAC2: Histone deacetylase 2
IQ: Intelligence quotient
IL- 6: Interleukin-6
IL- 1β: Interleukin-1β
lgG: Immunoglobulin G
MCT: Medium chain triglyceride
MMP- 9: Matrix metalloproteinases
NAA: N-acetylaspartate
PFC: Prefrontal cortex
PUFA: Polyunsaturated fatty acids
ROS: Reactive oxygen species
SRS: Social sensitivity scale
TBHQ: Tert-butylhydroquinone
tCr: Total creatine
tNAA: Total N-acetylaspartate
TNF-α: Tumor necrosis factor-α
UVB: Ultraviolet B
VDR: Vitamin D receptor
18 FDG PET: 18 Fluoro-deoxyglucos
