# Supplementary material for: Nutritional Approaches in Autism Spectrum Disorder: A Scoping Review
Source: Curr Nutr Rep. 2025 Apr 22;14(1):61. doi: 10.1007/s13668-025-00655-y (PMC12011661; doi:10.1007/s13668-025-00655-y)
Supplement: Supplementary file 1 — Supplementary file1 (PDF 200 KB) [file 13668_2025_655_MOESM1_ESM.pdf]

YAKAMOZ çeviri

Certificate of Proofreading

This document certifies that the manuscript listed below was proofread for proper grammar, spelling, punctuation, and overall style by Emel Zindan De Camillis, one of *Yakamoz Translation Bureau's* English-speaking editors/proof-readers:

Manuscript Title: [NUTRITIONAL APPROACHES IN AUTISM SPECTRUM DISORDER: A SCOPING REVIEW]

Author(s): [Prof. Dr. Nevin Şanlıer**]**

Date: May 2, 2024

YAKAMOZ TRANSLATION BUREAU

YAKAMOZ TERCÜME OFİSİ
Emel Zindan De Camillis
1683 Sk. No:47/13 Karşıyaka - İZMİR
Karşıyaka V.D. T.C. 100 829 657 08
Tel: 0232 464 79 65
